# Short-term safety of adjuvant chemoradiotherapy after local resection for patients with high-risk submucosal invasive rectal cancer: a single-arm, multicenter phase II trial

**DOI:** 10.1093/jjco/hyaa260

**Published:** 2021-02-09

**Authors:** Masaaki Noguchi, Kohei Shitara, Akihito Kawazoe, Daisuke Yamamoto, Yasumasa Takii, Yutaka Saito, Toshihiko Sato, Takahiro Horimatsu, Hideki Ishikawa, Yoshinori Ito, Masaaki Ito, Hiroaki Ikematsu

**Affiliations:** Division of Gastroenterology and Hepatology, Department of Internal Medicine, The Jikei University School of Medicine, Tokyo, Japan; Department of Gastroenterology and Gastrointestinal Oncology, National Cancer Center Hospital East, Chiba, Japan; Department of Gastroenterology and Gastrointestinal Oncology, National Cancer Center Hospital East, Chiba, Japan; Department of Gastroenterological Surgery, Ishikawa Prefectural Central Hospital, Ishikawa, Japan; Department of Gastroenterological Surgery, Niigata Cancer Centre Hospital, Niigata, Japan; Endoscopy Division, National Cancer Center Hospital, Tokyo, Japan; Department of Surgery, Yamagata Prefectural Central Hospital, Yamagata, Japan; Department of Therapeutic Oncology, Graduate School of Medicine, Kyoto University, Kyoto, Japan; Department of Molecular-Targeting Cancer Prevention, Graduate School of Medical Science, Kyoto Prefectural University of Medicine, Kyoto, Japan; Department of Radiation Oncology, Showa University School of Medicine, Tokyo, Japan; Department of Colorectal Surgery, National Cancer Center Hospital East, Chiba, Japan; Department of Gastroenterology and Endoscopy, National Cancer Center Hospital East, Chiba, Japan

**Keywords:** adjuvant chemoradiotherapy, rectal cancer, capecitabine, local resection

## Abstract

**Background:**

Surgery is recommended for patients with high-risk submucosal invasive rectal cancer (SM-RC) after local resection but affects the quality of life due to stoma placement or impaired anal function; therefore, alternative treatment approaches are needed to prevent local metastasis. The purpose of this study was to assess the short-term safety of adjuvant chemoradiotherapy with capecitabine in patients with high-risk submucosal invasive rectal cancer after local resection.

**Methods:**

This single-arm, multicenter, phase II trial included patients undergoing local resection for high-risk submucosal invasive rectal cancer within 12 weeks prior to enrollment. High-risk submucosal invasive rectal cancer was defined as the presence of at least one of the following factors: poor differentiation of adenocarcinoma, submucosal invasion depth > 1 mm, presence of lymphovascular invasion and grade-2 or -3 tumour budding. Protocol treatment comprised 45.0 Gy radiotherapy with conventional fractionation and 1650 mg/m^2^ capecitabine given twice daily until radiotherapy completion. The primary endpoint was treatment completion rate with an expected rate of 95% and a threshold of 80%.

**Results:**

Twenty-nine patients from six institutions were enrolled between May 2015 and February 2018. One patient was ineligible. Twenty-three patients completed treatment, with a completion rate of 82% (80% confidence interval, 69–91%); the remaining five patients completed treatment with protocol deviation. The median relative dose intensity of capecitabine was 100% (range, 58–100%). Common adverse events included radiation dermatitis (54%), anal pain (39%) and anal mucositis (29%). No grade-3 or higher adverse events were reported.

**Conclusions:**

Adjuvant chemoradiotherapy using capecitabine demonstrated acceptable short-term safety profiles in patients with high-risk submucosal invasive rectal cancer after local resection.

## Introduction

Due to the challenges in the definitive determination of invasion depth in submucosal invasive colorectal cancer (SM-CRC) by endoscopy before treatment, local resection, including endoscopic resection, is sometimes performed as an excisional biopsy. After local resection, patients with SM-CRC who exhibit specific histological features including poorly differentiated/mucinous adenocarcinoma, signet ring-cell carcinoma, a submucosal invasion depth of >1 mm, lymphovascular invasion and grade-2 or -3 tumour budding are at high risk of developing lymph node metastasis (1,2). The 2019 guidelines on the treatment of colorectal cancer published by The Japanese Society for Cancer and of the colon and rectum recommend additional surgery in patients with one or more of the abovementioned high-risk features confirmed by histological diagnosis (3). The standard additional surgery in patients with high-risk submucosal invasive colon cancer (SM-CC) and high-risk submucosal invasive rectal cancer (SM-RC) is colectomy and total mesorectal excision (TME), respectively. Complications of TME, including anal, urinary and sexual dysfunction, have a negative impact on the quality of life (QoL) compared with colectomy (4–7).

We previously reported that the rates of recurrence in patients undergoing local resection alone for high-risk SM-CC and SM-RC were 1.4% and 16.2% (*P* < 0.01), respectively. In addition, the local recurrence rates after radical surgery for high-risk SM-CC and SM-RC were 0.3% (1/376) and 0.6% (1/156), respectively. Furthermore, the rate of lymph node metastasis in radial surgery for high-risk SM-RC was 10.9%. Meanwhile, patients with high-risk SM-RC are more prone to refusing additional surgery than those with high-risk SM-CC (8), probably because of the negative impact of surgery on QoL. Thus, the development of alternative adjuvant treatments is warranted to prevent local metastasis in patients with high-risk SM-RC.

A systematic review of rectal cancer revealed that postoperative radiotherapy (RT) reduced local recurrence compared with surgery alone (9). In a randomized controlled trial (RCT) comparing postoperative chemoradiotherapy (CRT) using 5-FU with RT for locally advanced rectal cancer (LARC), CRT reduced local recurrence and cancer-related deaths (10). Another RCT provided evidence that preoperative CRT for T3 or more rectal cancer improved local control as compared with postoperative CRT (11). Therefore, adjuvant CRT for SM-RC with high-risk histological features after local resection may provide local control and may be considered as an adjuvant treatment option. In a Japanese prospective study, adjuvant CRT including 45.0 Gy RT and continuous 5-week infusional administration of 5-fluorouracil (5-FU) in patients with high-risk T1 low rectal cancer after local resection exhibited favourable outcomes (5-year disease-free survival rate, 94%) and a high degree of safety (12).

Compared with infusional administration of 5-FU, capecitabine, which is an orally administered fluoropyrimidine, is more convenient and equally effective when used in combination with RT for LARC (13,14). Thus, we performed a phase II trial to evaluate the short-term safety and efficacy of adjuvant CRT using capecitabine in patients with high-risk SM-RC after local resection.

## Patients and methods

### Study design and patients

This was a single-arm, multicenter phase II trial involving six institutions in Japan. Eligible patients were 20–80 years of age and had an Eastern Cooperative Oncology Group performance status score of 0 or 1. All patients had been diagnosed with early rectal cancer, had undergone local resection within 12 weeks before trial enrollment and had histologically confirmed *en-bloc* resection of high-risk SM-RC, with negative lateral and vertical margins. Local resection approaches included polypectomy, endoscopic mucosal resection, endoscopic submucosal dissection, peranal local excision, transanal endoscopic microsurgery and minimally invasive transanal surgery. High-risk SM-RC was defined as pathological submucosal cancer satisfying one or more of the following criteria: poorly differentiated/mucinous adenocarcinoma, signet ring-cell carcinoma, a submucosal invasion depth of >1 mm, lymphovascular invasion and grade-2 or -3 tumour budding. Eligible patients had no history of receiving chemotherapy or RT; had adequate bone marrow, hepatic and renal function as evidenced by blood tests; and refused further surgery despite the fact that surgery is recommended as standard therapy. The absence of lymph node and distant metastases was confirmed by computed tomography within 16 weeks before enrollment. Patients with other active malignancies and those with a history of steroid treatment, active viral hepatitis, active infection or psychiatric diseases were ineligible since these factors and conditions could increase the risk associated with study participation.

The study protocol was approved by the institutional review boards at each participating site, and the trial was performed in accordance with the ethical principles of the Declaration of Helsinki. All patients provided written informed consent.

This study was registered with the University Hospital Medical Information Network (number UMIN000016785).

### Procedures

The total irradiation dose of 45.0 Gy was delivered in 25 fractions (1.8-Gy daily fractions administered over a period of 5 weeks, excluding holiday or weekends) in combination with orally administered capecitabine. Capecitabine at 1650 mg/m^2^ was administered continuously twice daily for the whole RT treatment period (13,15,16).

RT was delivered with a megavoltage equipment (>6 MV), in which the source-surface distance and source-axis distance was ≥100 cm. RT required computed tomography-based treatment planning. The clinical target volume (CTV) was defined as the primary tumour bed and mesorectum. The cranial CTV border corresponded to the level of the recto-sigmoid junction, and the caudal CTV border was 2 cm below the lowest tumour border. The anterior CTV border was the axial section containing the prostate, seminal vesicles, posterior bladder wall, uterus or vagina and required a 10-mm margin anterior from the bladder, seminal vesicles or uterus to account for the variations in bladder volume. The planning target volume was defined as the CTV plus an appropriate margin (0.5–1.0 cm) to account for internal organ movements and daily setup errors.

### Outcomes

The primary endpoint was treatment completion rate, which was defined as fulfilment of both of the following criteria: (i) completion of RT within 21 days from the planned date, (ii) capecitabine was administered for ≥75% of the planned number of days. Secondary endpoints included adverse events, relative dose intensity (RDI) and relapse-free survival. The treatment completion rate, RDI and relapse-free survival were analyzed based on the full analysis set, and adverse events were analyzed based on the safety analysis set. The severity of adverse events during the protocol treatment was graded according to the National Cancer Institute-Common Terminology Criteria for Adverse Events (version 4.0) (17). Relapse-free survival was defined as the period from the time of enrollment until the day of relapse or death from any cause, whichever was earlier. RDI was calculated as the proportion of the actually received total dose of capecitabine compared with the defined total dosage of capecitabine. The defined total dosage was calculated by multiplying the actual number of treatment days with the initial daily dosage.

### Statistical analysis

Descriptive statistics were used to analyze the data. A sample size of 35 patients was calculated based on an expected completion rate of 95% and a threshold of 80%, with a one-sided α of 10% and a power of 80%. For completion rate, 80% confidence intervals were calculated according to the exact binomial distribution (18,19).

## Results

### Patient characteristics

A total of 29 patients from the six participating institutions were enrolled between May 2015 and February 2018. The study was discontinued because of slower accrual than expected. One patient was ineligible due to concurrent steroid administration. The characteristics of the remaining 28 patients are summarized in Table 1. Briefly, the median age was 67 (range, 33–77) years, and 64% of the patients were males. All patients had an Eastern Cooperative Oncology Group PS score of 0. The majority of the patients (75%) had rectal cancer below the peritoneal reflection. Seven (25%) patients underwent transanal resection. Regarding histological factors associated with high risk, 26 (95%) patients had a submucosal invasion depth of >1 mm, 10 (36%) patients had lymphovascular invasion, 7 (25%) patients had a tumour budding grade of 2 or 3, and none of the patients had poorly differentiated/mucinous adenocarcinoma or signet ring-cell carcinoma. Overall, 16 (57%), 9 (32%) and 3 (11%) patients had one, two and three histological high-risk factors, respectively. The median interval from resection to CRT was 58 (29–99) days.

**Table 1:** Patients’ characteristics

	*N* = 28	%
Age		
Median (range), years	67 (33–77)	
Sex		
Male	18	64
Female	10	36
ECOG PS score		
0	28	100
Location		
Ra	7	25
Rb	21	75
Method		
EMR	8	29
ESD	13	46
PAE	5	18
TEM	2	7
Tumour size		
Median (range), mm	18 (8–55)	
Macroscopic type		
Is	14	50
Isp	5	18
IIa	9	32
Differentiation of tumour		
Well	20	71
Moderate	8	29
Factor of high risk		
Poorly differentiated/mucinous adenocarcinoma signet ring-cell carcinoma	0	0
Submucosal invasion depth > 1 mm	26	93
Lymphovascular invasion	10	36
Budding grade of 2 or 3	7	25
Interval from resection to CRT		
Median (range), days	58 (29–99)	

ECOG PS, Eastern Cooperative Oncology Group performance status; Ra, rectum above peritoneal reflection; Rb, rectum below peritoneal reflection; EMR, endoscopic mucosal resection; ESD, endoscopic submucosal dissection; PAE, peranal local excision; TEM, transanal endoscopic microsurgery; CRT, chemoradiotherapy.

**Table 2:** Treatment results

		*N* = 28	Proportion
Treatment completion	Yes	23	82% (80% CI, 69–91%)
Radiotherapy			
Received total dose	Yes	28	100%
Completion within 21 days from the planned date	Yes	28	100%
Chemotherapy			
≥75% of the planned number of days	Yes	23	82%
	No	5	18%
	Protocol deviation	5	18%
	AEs	0	0%
Relative dose intensity	Median		100% (range, 58–100%)

AE, adverse event; CI, confidence interval.

### Treatment results

 Treatment results were shown in Table 2. All 28 patients received a total dose of 45.0 Gy RT divided into 25 fractions and completed RT within 21 days from the planned date. Additionally, 23 (82%) patients were administered capecitabine for ≥75% of the planned number of days. The remaining five (18%) patients did not receive capecitabine enough days due to protocol deviation from the treatment schedule and were treated with capecitabine for 5 days per week. The reasons for these deviations were a misunderstanding of the schedule of the protocol treatment.

Of the 28 patients including five patients who deviated from the protocol treatment, 23 patients completed the protocol treatment, with a completion rate of 82% (80% confidence interval, 69–91%). The median RDI of capecitabine was 100% (range, 58–100%).

### Adverse events

The adverse events observed in the study cohort are summarized in Table 3. The most common adverse events were radiation dermatitis (54%), anal pain (39%) and anal mucositis (29%). All 28 patients completed RT without any interruption due to adverse events. One patient (4%) discontinued capecitabine due to drug-related toxicity (grade-2 hand-foot syndrome), although this patient met the criteria for treatment completion (>75% of the planned dose received). The remaining 27 (96%) patients were administered capecitabine treatment until the end of RT. No one reduced the dosage of capecitabine and delayed the protocol treatment due to adverse events. No adverse events of grade 3 or higher were observed.

**Table 3:** Adverse events

Adverse events, *N* = 28	Any grade (%)	Grade 1 (%)	Grade 2 (%)	Grade ≥ 3 (%)
Any events	25 (89)	14 (50)	11 (39)	0 (0)
Hematological	14 (50)	7 (25)	7 (25)	0 (0)
Anemia	7 (25)	7 (25)	7 (25)	0 (0)
Neutropenia	7 (25)	4 (14)	3 (11)	0 (0)
Thrombocytopenia	2 (7)	2 (7)	0 (0)	0 (0)
Non-hematological	24 (86)	19 (68)	5 (18)	0 (0)
Nausea	2 (7)	2 (7)	0 (0)	0 (0)
Diarrhea	6 (21)	5 (18)	1 (4)	0 (0)
Anal mucositis	8 (29)	3 (11)	5 (18)	0 (0)
Anal pain	11 (39)	9 (32)	2 (7)	0 (0)
Fatigue	3 (11)	3 (11)	0 (0)	0 (0)
Anorexia	3 (11)	3 (11)	0 (0)	0 (0)
Hand-foot syndrome	4 (14)	4 (14)	0 (0)	0 (0)
Cystitis noninfective	3 (11)	2 (7)	1 (4)	0 (0)
Dermatitis radiation	15 (54)	13 (46)	2 (7)	0 (0)

### Relapse-free survival

Relapse-free survival was not evaluated at the time of this preliminary analysis.

## Discussion

The present phase II trial showed that adjuvant CRT using capecitabine demonstrated acceptable short-term safety profiles in patients with high-risk SM-RC after local resection. To the best of our knowledge, this is the first phase II trial to evaluate the safety of adjuvant CRT with capecitabine in patients with high-risk SM-RC after local resection.

In the present study, the treatment completion rate of 82% was similar to the previously reported prospective studies. In a Japanese study of adjuvant CRT comprising 45.0 Gy RT with continuous infusional administration of 5-FU after local resection in patients with T1–T2 low rectal cancer satisfying one or both of the following features: submucosal invasion depth > 1 mm and lymphovascular invasion, the treatment completion rate was 86% (12).

The median RDI of capecitabine was 100%. These findings suggest that most patients can be administered capecitabine as planned without interruption due to adverse events. Although we applied continuous administration of capecitabine based on a previous phase 3 trial (13), five (18%) patients received capecitabine for only 5 days per week, reflecting protocol deviation from the treatment schedule, which was also reported in another study (20).

Although grade-1 and -2 adverse events were common in the present study (89%), adverse events of grade 3 or higher were not observed, and the most common adverse events were radiation dermatitis, anal pain, and anal mucositis.

The toxicities observed in the present study were similar to those in the Japanese study of adjuvant CRT after local resection but lower than those in the neoadjuvant CRT for LARC (NSABP R-04 trial). In the Japanese study of adjuvant CRT comprising 45.0 Gy RT with continuous infusional administration of 5-FU in patients with high-risk T1–T2 rectal cancer after local resection, 20 of 57 (35%) patients developed CRT-related adverse events, and the most common adverse events were diarrhea (11%) and anal pain (11%) (12). Meanwhile, the NSABP R-04 trial reported that in the capecitabine without oxaliplatin group, adverse events of grade 3 or higher and diarrhea occurred in 39.0% and 17.1% of the patients, respectively (20). The high toxicities in the NSABP R-04 trial may be due to the different periods of CRT because neoadjuvant CRT for LARC requires an additional boost dose of 5.4 Gy to the tumour bed.

The current study has several limitations. First, the study sample size was small and did not reach the planned sample size, thus the statistical power was reduced. Second, the protocol deviation from the treatment schedule might have led to reductions in the treatment completion rate and the frequency of adverse events. Finally, the present study evaluated the short-term safety but not the efficacy of the treatment. Therefore, our group is currently performing a single-arm confirmatory trial of adjuvant CRT for patients with high-risk SM-RC after local resection, which is registered with the Japan Registry of Clinical Trials (jRCT1031180076). A Dutch group is also currently performing a multicenter randomized trial of radical surgery vs*.* adjuvant CRT after local excision for high-risk T1 and low-risk T2 rectal cancer (21).

## Conclusions

In conclusion, adjuvant CRT using capecitabine demonstrated acceptable short-term safety profiles in patients with high-risk SM-RC after local resection. Further studies should aim at evaluating the efficacy of the protocol treatment, which is expected to become an alternative approach for patients with high-risk SM-RC after local resection.

## Acknowledgments

We would like to express our gratitude to Ms. Naoko Sawada from Medical Research Support Co., Ltd for assisting with the data management. We thank all patients included in this study, their families and all physicians.

## Conflict of interest statement

Kohei Shitara reports paid consulting or advisory roles for Astellas, Eli Lilly, Bristol-Myers Squibb, Takeda, Pfizer, Ono Pharmaceutical, MSD, Taiho, Novartis, AbbVie, and GSK; honoraria from Novartis, AbbVie, and Yakult; and research funding from Astellas, Eli Lilly, Ono Pharmaceutical, Sumitomo Dainippon Pharma, Daiichi Sankyo, Taiho, Chugai, MSD, and Medi Science, outside the submitted work. Kawazoe Akihito reports honoraria from Taiho Pharmaceutical, Ono Pharmaceutical and Bristol-Myers Squibb; and research funding from Taiho Pharmaceutical, Ono Pharmaceutical, Sumitomo Dainippon Pharma and MSD, outside the submitted work. Masaaki Ito reports personal fees from Yakult Honsha, Covidien Japan, Chugai Pharmaceutical, Johnson & Johnson K.K. Medical Company, Olympus Corporation, Medical Leaders, Taiho Pharmaceutical, Applied Medical Japan, Miyarisan Pharmaceutical, Sanofi K.K, Tsumura & Co. and Daiichi Sankyo; and grants from Johnson & Johnson K.K. Medical Company, Muranaka Medical Instruments, Fujita Medical Instruments, Mitsubishi Precision, Kawasumi Laboratories, Intelligent Surfaces, A1 Technica, Akita Sumitomo Bakelite, outside the submitted work; In addition, Dr. Ito has a patent Muranaka Medical Instruments with royalties paid, and a patent EBM Corporation with royalties paid. All other authors have no conflicts of interest to declare.

## Funding

This work was supported by the National Cancer Center Research and Development Fund (25-A-12) awarded to Dr Saito.
